# Familial acute myeloid leukemia due to a novel germline CEBPA pathogenic variant – a case report

**DOI:** 10.1016/j.htct.2024.07.004

**Published:** 2024-09-12

**Authors:** Flavia Gava, Luiz Fernando Bazzo Catto, Elvis Valera, Maristella Bergamo Francisco dos Reis, Maria Carolina Tostes Pintão, Maria de Lourdes Chauffaille, Flavia Sacilotto Donaires Ramos, Carlos Alberto Scrideli, Lorena Lobo Figueiredo Pontes

**Affiliations:** a Department of Pediatrics, Ribeirão Preto Medical School, University of São Paulo, Ribeirão Preto, São Paulo, Brazil; b Department of Medical Images, Hematology, and Clinical Oncology, Ribeirão Preto Medical School, University of São Paulo, Ribeirão Preto, São Paulo, Brazil; c Fleury Group Laboratory, São Paulo, Brazil

## Introduction

Myeloid neoplasms associated with germline predisposition are rare entities that were recently included by the World Health Organization (WHO) as a distinct category of hematological malignancies.^1^ These neoplasms can be classified into (1) neoplasms without a preexisting disorder (due to *CEBPA, DDX41* and *TP53* variants), (2) neoplasm with other organ dysfunction (GATA2, telomere disease, RASopathies, Down syndrome, *SAMD9, SAMD9L*, biallelic germline BLM and bone marrow failure syndromes such as Fanconi anemia, Shwachman-Diamond syndrome and severe congenital neutropenia), and (3) neoplasm with a history of thrombocytopenia (*RUNX1, ETV6*, and *ANKRD6* variants).^1^^,^^2^

*CEBPA* is a single exon gene located in the 19q13.1 chromosomal region. It encodes the CCAAT/enhancer-binding protein, the expression of which is confined to myeloid cells, and is crucial for normal myeloid differentiation.^1^^,^^3^, ^4^, ^5^, ^6^

CEBPA mutations, described in 5–15 % of acute myeloid leukemia (AML) patients,^1^ can be categorized into three types: 1) CEBPA single mutation (*CEBPAsm*), one mutation in one allele, 2) *CEBPA* double mutation (*CEBPAdm*), an N-terminal mutation and a basic leucine zipper motif mutation, and 3) CEBPA homozygous mutation due to loss of heterozygosity. Irrespective of the occurrence as biallelic or monoallelic mutations, it is now considered that in-frame mutations affecting the basic leucine zipper region of *CEBPA* confer favorable prognosis according to the most recent European Leukemia-Net (ELN) recommendations.^7^

One study with a cohort of 187 patients with AML detected 18 patients (9.6 %) with *CEBPA* mutations.^3^ In this cohort, two patients carried germline *CEBPA* mutations, indicating that somatic mutations are a frequent secondary event in *CEBPA*-associated familial AML.^3^ In fact, around 10 % of *CEBPAdm* cases have a germline hit with a second somatic mutation at a different locus.^4^

The first family with a germline *CEBPA* mutation, in which the father and his two children developed AML within two weeks, was described in 2004.^8^ Germline *CEBPA* mutations are rare with the largest cohort published evaluating ten families.^4^ This study demonstrated highly penetrant predisposition to develop AML, and the absence of clinical features preceding AML diagnosis. Furthermore, despite the good prognosis, AML recurrence is high, with approximately 50 % of patients developing relapse after prolonged remission.^1^^,^^4^ Additionally, all patients with *CEBPA*-associated familial AML carried a frameshift mutation in the N-terminal region, and a somatic mutation in the C-terminal region.^1^ Here, we describe a new germline pathogenic variant in a *CEBPA*-associated familial AML case.

## Case report

**Figure 1:**
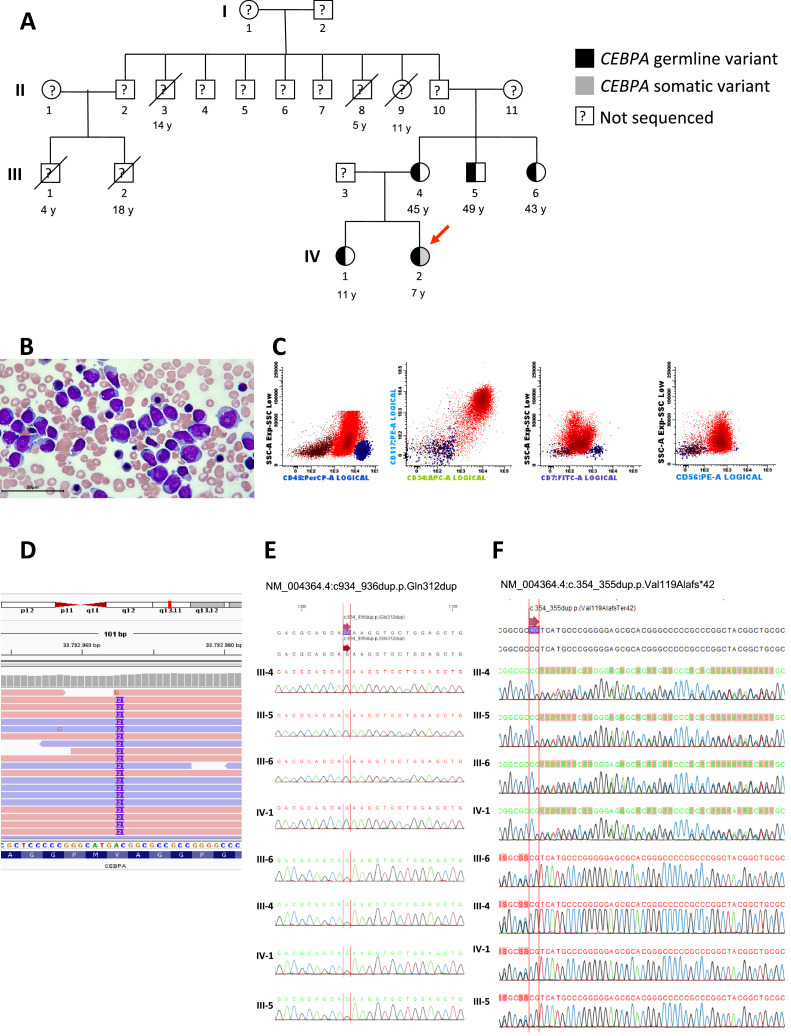
Phenotypic and genotypic characterization of an inherited CEBPA germline mutation in a case of pediatric acute myeloid leukemia. (A) The heterozygous germline CEBPA (p.Val119Alafs*42) variant in pedigrees is shown in black. The index case carrying a somatic variant (gray square) is highlighted (red arrow). (B) Morphology of blasts in bone marrow smear (Giemsa stain). (C) Immunophenotyping of bone marrow demonstrating the expression of CD56 and CD7 in blast cells. (D) Integrative genomic view (IGV) of CEBPA gene in the index case. (E) Chromatograms demonstrate the absence of somatic variant in CEBPA in III-4, III-5, III-6 and IV-1 (F) Chromatograms demonstrate germline variant in CEBPA in III-4, III-5, III-6 and IV-1.

A 6-year-old female patient was admitted with a one-month history of recurrent fever associated with pallor, weakness, fatigue, petechiae, and bruising. Family history revealed five maternal family members diagnosed with AML at 14, 11, 5, 4, and 18 years old (Figure 1A, individuals II-3, II-8, II-9, III-1 and III-2 respectively). Physical examination showed cutaneous pallor and diffuse petechiae. Peripheral blood count revealed hemoglobin concentration of 9.1 g/dL, leukocytes of 3600/μL, neutrophils of 400/μL, lymphocytes of 1900/μL, platelets of 9000/μL and 21 % of blasts. Bone marrow morphology and flow cytometry analysis revealed 57 % of blasts (Figure 1B) which expressed MPO, CD34, CD117, CD33, CD13, HLA-DR, CD133, CD7, and CD56 (Figure 1C). Bone marrow karyotype was 46,XX[20].

Whole-exome sequencing (WES) of a blood sample from the index case (IV-2) at diagnosis demonstrated two variants in *CEPBA* (NM_004364.4). One was a germline pathogenic variant at NM_004364.4:c.354_355dup.p.Val119Alafs*42 with a variant allele frequency (VAF) of 45 %, and the other was a somatic likely pathogenic variant at NM_004364.4:c934_936dup.p.Gln312dup with a VAF of 38 %, both in heterozygosis (Figure 1D, 1E, and 1F). No other pathogenic germline or somatic variants were found. Both variants are not described in the population genome database gnomAD ^9^ or the variant databases dbSNP and ClinVar hosted by the National Center for Biotechnology Information (NCBI, NIH, USA). According to the most recent classifications, the case was categorized as *CEBPA*-associated familial AML (WHO) and AML with germline *CEBPA* mutation (International Consensus Criteria, ICC).^10^^,^^11^

Buccal mucosal specimens from the mother, two uncles, and sister were submitted to Sanger sequencing for both *CEBPA* variants revealing only the germline variant c.354_355dup (p.Val119Alafs*42) in heterozygosis (Figure 1E, and 1F). No blood cell count abnormalities were found in the tested relatives (Table 1).

**Table 1:** Blood cell counts of the affected relatives of the index case with the germline CEBPA (p.Val119Alafs*42) variant.

Generation	Age (years)	Hb (g/dL)	MCV (FL)	WBC (10^3^/µL)	Neutrophils (10^3^/µL)	Platelets (10^3^/µL)
**III-4**	45	12.2	95	4300	2967	161,000
**III-5**	49	13.8	80	7900	5000	205,000
**III-6**	49	12.4	94	4700	2600	258,000
**IV-1**	11	15.9	88	13,000	6491	357,000

Hb: Hemoglobin; MCV: Mean corpuscular volume; WBC: White blood cell count.

The patient was treated according to the Childhood Acute Myeloid Leukemia Study Group (GELMAI) protocol ^12^ and achieved complete remission, with negative minimal measurable residual disease by flow cytometry after remission induction and consolidation therapies. The child has been clinically followed post-treatment, without bone marrow transplantation (BMT), and remains in complete remission two years after treatment.

## Discussion

This report addresses the diagnostic work-up and management of an AML case involving a new germline pathogenic variant of the *CEBPA* gene. Unlike other familial predisposition syndromes, familial *CEBPA* AML typically presents without abnormalities in blood count (Table 1).^1^ Additionally, as described in the current case and in previous studies, the blast characteristically expresses CD7 (Figure 1C).^4^

WES analysis demonstrated that the present patient had two variants in *CEBPA,* one was a germline pathogenic variant and the other, a somatic likely pathogenic variant. No more pathogenic abnormalities were found. A second somatic mutation in *CEPBA* is common in patients with germline CEBPA associated leukemia.^4^^,^^13^ Additionally, as previously described, the presence of a germline *CEBPA* variant might impact the selection of particular cooperating mutations, with the susceptibility to mutation acquisition possibly influenced by inherited factors that are frequently shared among family members.^4^

Somatic mutations in *CEBPA, GATA2, TET2*, and *WT1* were associated with clonal evolution to AML in patients with germline *CEBPA* variants.^13^ However, as we described above, we only found a second somatic mutation in the *CEBPA* gene.

Despite the high response rate in germline *CEBPA* AML, the cumulative incidence of relapse in this setting is high with multiple and late recurrences having been described.^3^^,^^4^^,^^14^, ^15^ BMT is potentially curative, but there is no consensus about BMT in this setting. Screening for genetic variants prior to the selection of sibling donors is essential to identify asymptomatic mutation carriers, as is adequate family follow-up and genetic counseling.^4^ In this case, a histocompatibility study was performed, and her sister was fully matched, but carried the same germline *CEBPA* variant. Despite finding an unrelated-matched donor, we opted against BMT based on some factors, such as age, possibility of a prolonged period of remission, morbidity related to transplant, and complete response with chemotherapy. The patient remains in complete remission after two years of rigorous clinical follow-up.

In conclusion, germline predisposition is an important qualifier of AML that influences patient management, bone marrow donor selection, choice of conditioning regimen for transplant, and genetic counseling. This case highlights the need to recognize germline mutations in AML, especially in children without a constitutional disorder affecting other organ systems. Both AML diagnosis early in life as well as the strong familial history of hematopoietic neoplasms must prompt consideration for such conditions. In addition, among the *CEBPA* variants already described, the novel variant reported here will contribute to the knowledge of this rare and intriguing entity.

Original data and detailed methods are available by email request to the corresponding author.

## Conflicts of interest

None.
